# Tiny Microbes, Big Yields: enhancing food crop production with biological solutions

**DOI:** 10.1111/1751-7915.12804

**Published:** 2017-08-25

**Authors:** Pankaj Trivedi, Peer M. Schenk, Matthew D. Wallenstein, Brajesh K. Singh

**Affiliations:** ^1^ Bioagricultural Science and Pest Management Colorado State University Fort Collins CO 80523 USA; ^2^ School of Agriculture and Food Sciences The University of Queensland Brisbane QLD 4072 Australia; ^3^ Department of Ecosystem Science and Sustainability Colorado State University Fort Collins CO 80523 USA; ^4^ Natural Resource Ecology Laboratory Colorado State University Fort Collins CO 80523 USA; ^5^ Hawkesbury Institute for the Environment Western Sydney University Richmond NSW 2753 Australia; ^6^ Global Center for Land Based Innovation Western Sydney University Richmond NSW 2753 Australia

## Abstract

Plant‐associated microbiomes have tremendous potential to improve plant resilience and yields in farming systems. There is increasing evidence that biological technologies that use microbes or their metabolites can enhance nutrient uptake and yield, control pests and mitigate plant stress responses. However, to fully realize the potential of microbial technology, their efficacy and consistency under the broad range of real‐world conditions need to be improved. While the optimization of microbial biofertilizers and biopesticides is advancing rapidly to enable use in various soils, crop varieties and environments, crop breeding programmes have yet to incorporate the selection of beneficial plant–microbe interactions to breed ‘microbe‐optimized plants’. Emerging efforts exploring microbiome engineering could lead to microbial consortia that are better suited to support plants. The combination of all three approaches could be integrated to achieve maximum benefits and significantly improved crop yields to address food security.

## Sustainable development goals and agriculture productivity

Sustainable increases in agricultural productivity are critical to address multiple Sustainable Development Goals (SDGs) including zero hunger (SDG 2), no poverty (SDG 1) and good health and well‐being (SDG 3). Increased productivity can also significantly contribute to various other SDGs including SDG 6 (clean water and sanitation), SDG 9 (industry, innovation and infrastructure), SDG 13 (climate Action) and SDG 15 (life on land). To meet the food requirement for a global population exceeding 9 billion by 2050, crop productivity needs to increase by 70–100%. Conventional intensive agricultural practices that depend on inorganic fertilizers, pesticides and other chemical inputs have increased yield but also contributed to soil degradation, loss of biodiversity, increased susceptibility of crops to pests/pathogens and negative environmental impacts which, together, have significant consequences for human health and food security (Tilman *et al*., 2002). In addition to structural decline in farm productivity (where further increase in inputs does not result in proportional yield increases) in developed countries, major challenges in developing countries lie in substantially increasing yield quality and quantity, without further increases in farming costs and detrimental environmental impacts.

**Figure 1:**
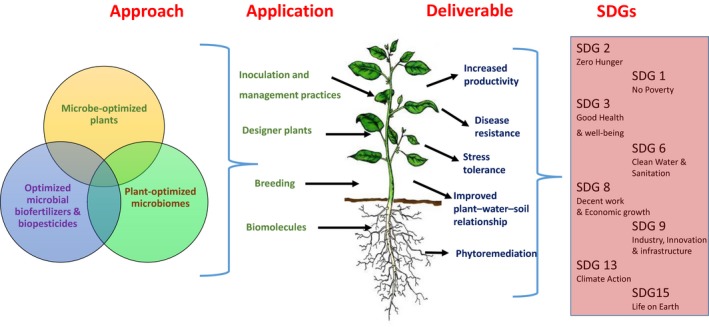
Sustainable increase in farm productivity by harnessing microbial technologies is critical for delivery of multiple Sustainable Development Goals (SDGs). It will primary contribute to SDGs 2 (by increasing farm productivity) and 1 (by increasing farm profitability) but will also significantly contribute to SDGs 3, 6, 13 and 15 by improving nutrient quality, reducing environmental chemical pollutions, reducing greenhouse gas emission and promoting soil biodiversity respectively. SDGs 8 and 9 will benefit from creating of new industry for the production of microbial products and formulation. The cartoons of individual SDGs were obtained from UN SDGs web page (http://www.un.org/sustainabledevelopment/sustainable-development-goals/).

It is clear that expansion of conventional agricultural practices to meet future demands is neither economically nor environmentally feasible. There is an urgent need for complimentary approaches to sustainably meet the global food security demands. One way to develop improved and advanced sustainable crop production method is to enhance the beneficial plant‐associated microbiome. Microbes have the potential to increase crop growth and vigour, nutrient use efficiency, biotic/abiotic stress tolerance and disease resistance (Figure 1). If this potential can be harnessed under real‐world conditions, it could improve farm productivity and food quality in a sustainable manner, leading to positive environmental, social and economic outcomes.

## Technical challenges and emerging solutions

Microbial products can increase crop yields and have potential to complement or replace agricultural chemicals and fertilizers. Many companies have started to exploit individual microorganisms as biocontrol or biofertilizer products and develop carrier‐based inoculants of beneficial strains. The results from large‐scale field trials have demonstrated an increase of 10–20% in crop production on economically important crop plants (Pérez and Trivedi, 2017; Pérez‐Jaramillo *et al*., 2016). Despite the high potential of microbial technologies, available evidence suggests that encouraging results from greenhouse trials often fail to manifest in field trials. The effects of microbial products are often inconsistent between different studies and vary with climatic/edaphic conditions, which is the major bottleneck in the large‐scale adoption of the technology. Therefore, there is an urgent need to improve the selection process and application technique and particularly to better understand the interactions between inoculated strains and native microbiomes under field conditions (Fig. 1).

The complexity of interactions among microbes, plants, soil and climate appears daunting. Until recently, these interactions have only been studied under simplified conditions. New technologies including microfluidics‐based technologies such as ‘Microbiome on a Chip’ will facilitate multiplexed treatments and incorporate environmental stimuli, host responses and the colonization of microbes, thus shedding light on multitrophic plant–microbiome interactions (Stanley and van der Heijden, 2017). Such knowledge can be used to model the desired activities of beneficial microbes in relevant field conditions or assess whether native microbiomes influence the beneficial activities and/or the colonization potential of the inoculated strains. This, in combination with improved application technologies and easy‐to‐use formulations with long shelf lives, will greatly improve the efficacy of microbial products in field conditions. One approach towards achieving this goal is to modify plant microbiomes and traits by introducing beneficial bacteria at flowering into the progeny seeds (Mitter *et al*., 2017). This mode of delivery has potential to introduce beneficial traits within one generation and has several advantages over conventional application techniques including better protection against competition from native microflora that significantly increases the colonization and survival potential of the inoculated strain.

Consortia of multiple compatible beneficial microbes that form associations with the rest of the microbiome, emulating strongly structured networks in natural rhizosphere soils, may have a better chance to survive and provide benefits to the host, compared with single‐strain formulations (Singh and Trivedi, 2017; Wallenstein, 2017). Through systematic isolation procedures, it is possible to capture the majority of the species in the natural communities present in the rhizosphere and phyllosphere (Bai *et al*., 2015). Similar approaches can expedite the isolation of ‘keystone’ microbial species and develop synthetic microbial communities that promote plant performance. Preliminary work suggests synthetic microbial communities can be successfully used to provide benefits to the plants in terms of early flowering, nutrient acquisition and disease resistance (Gopal and Gupta, 2016 and reference within).

Genotypic and phenotypic variations in plants select for different microbiomes. This suggests that the ability of a plant to support a beneficial microbiome is a plant trait under selection (Wallenstein, 2017). Domestication is postulated to remove this ‘microbiome‐mediated trait’ thus necessitating application of high quantities of inorganic fertilizers, spraying of insecticides and growth hormones to maintain the required output (Pérez‐Jaramillo *et al*., 2016). While the optimization of microbial biofertilizers and biopesticides is advancing rapidly to enable use in various soils, crop varieties and environments, crop breeding programmes have yet to incorporate the selection of beneficial plant–microbe interactions to breed ‘microbe‐optimized plants’. As the genetic make‐up of the plant plays a major role in the outcome of the beneficial plant–microbiome interactions, it is possible to breed for ‘designer plants’ that are optimized to attract and maintain beneficial microbes (Abhilash *et al*., 2012). Breeding programmes have only started exploring these traits, but before this can become a significant effort, a better understanding of how beneficial microbes are attracted and maintained by crops is essential. Genetic engineering and plant breeding would enable us to generate microbe‐optimized plants that produce the right exudates and volatiles to attract and maintain beneficial microbes at the right time, either at the root or on the leaf.

Plants engineer their own rhizosphere environment by the secretion of specific exudates to improve nutrient availability and interactions with specific beneficial microbes. A prerequisite for this is that the targeted microbes are present, so this strategy may need to be coupled with inoculation of corresponding microbes. In animal systems, microRNA (miRNA) modulates the colonization of specific microbes in guts and can restore healthy status (Liu *et al*., 2016). Preliminary work suggests a similar role for the miRNA in regulating the structure of rhizosphere microbiome. Therefore, elucidating the interactions between miRNA and the microbiome may provide a valuable toolkit to engineer a defined beneficial plant microbiome or transfer miRNA from a target soil to recipient soils for desirable outcomes.

Plant ecological engineering (e.g. integrating plant breeding with microbiome selection) has enormous potential to manipulate host microbiomes in order to enhance the effectiveness of disease management. Engineering plant/soil‐optimized microbes and plant/soil‐optimized microbiomes that can be used as inoculum for different crops in different soils can also be achieved by artificial ecosystem selection. While not yet applied in industrial settings, there is evidence that soil microbiomes adapt to their crops over time leading to improved plant–microbe interactions (Berendsen *et al*., 2012). A top‐down approach to improve animal and plant fitness by artificially selecting upon microbiomes, thus engineering evolved microbiomes with specific effects on host fitness, has been proposed (Mueller and Sachs, 2015). This host‐mediated microbiome engineering approach selects upon microbial communities indirectly through the host and leverages host traits that evolved to influence microbiomes. Evidence that microbiomes can be optimized for disease resistance by the application of phytohormones that activate defence responses has also been obtained (Lebeis *et al*., 2015). Along similar lines, optimized microbiomes that help plants develop early or flower later could be used as inoculants to provide drought resistance as plants are known to adopt altered flowering time in response to several abiotic stresses (Kazan and Lyons, 2016). Generating host‐mediated artificial selection of microbiomes may be a cheaper way to help curb plant diseases rather than pesticides and antibiotics, or creating genetically modified organisms. Furthermore, findings on the overlapping ‘functional core microbiome’ in different plant species provide strong support for cross‐compatibility of microbiome transfer with phylogenetically unrelated plant species. Sheth *et al*. (2016) have highlighted emerging *in situ* genome engineering toolkit to manipulate microbial communities with high specificity and efficacy over a range of specificities and magnitudes.

## Future perspective

In recent years, advancements in high‐throughput multi‐'omics' technologies and computational integration have helped us to understand plant–microbiome interactions across scales and decipher individual signal molecules, proteins, genes and gene cascades to connect them with functional gene networks/pathways. Technological advancements have facilitated the understanding of gene editing systems, RNAi‐mediated gene silencing, mutant technology and proteomics and metabolite profiling to reveal interactive networks that advanced our understanding of microbe‐mediated strategies of plant growth promotion and biocontrol. Advances in automation and large‐scale bioinformatics have increased the repertoire of available genomes of plant‐associated microbes and, together with information on their interactions with host/environment, are helping researchers to discover valuable new microbial genes for improved plant growth and productivity. These advances not only provide a resource and conceptual framework for studying plant–microbiome interactions, but also highlight many new potential plant‐beneficial genomic circuits that could be targeted to improve plant productivity around the globe. With an average of 5000 genes per organism and about 54000 complete genome sequences of microbes available in public domain, there is a vast library of over 250 million genes to be prospected for biotechnological applications.

Gene discoveries have resulted in the development of genetically engineered plants using novel microbial genes for disease resistance, herbicide tolerance, stress tolerance and plant yield improvement (Macdonald and Singh, 2014; and reference within). However, most of these breakthroughs were achieved by inserting a few genes or a combination of a few targets (multiplexing). Future research should focus on combining different strategies, such as the multigenic approach to simultaneously incorporate more than one gene in transgenic plants. New tools and resources that can be applied to introduce complex heterologous pathways into plants (Shih *et al*., 2016) hold the key to build synthetic genome clusters from microbiomes to enable the stacking and shuffling of disease resistance and stress tolerance traits between crop plants. New capabilities developed in trait discovery will further intensify the rate of novel gene discovery. For example, the CRISPR–Cas9‐based forward genetic screen will help future studies of plant–microbiome interactions to transcend individual genes and become more holistic in approaches to elucidate plant–microbiome interactions and discover novel genes for biotechnological applications (Barakate and Stephens, 2016).

A wealth of genome information dramatically expands our understanding of a variety of microbial metabolic pathways available for novel traits. This leads to attempts to design and engineer microbial cell factories devoted to elucidate and investigate new metabolic pathways, as well as the high‐level production of the respective compounds allowing their characterization and application. Potential applications exist in the field of sustainable plant cultivation, as several metabolites are known to improve plant health and growth. This can be effected via different mechanisms. Certain metabolites can, for instance, directly trigger enhanced plant growth as signal molecules; others can indirectly support plant growth by inhibition of plant pathogens or by shaping a beneficial microbiome around the plant.

The integration of microbial biofertilizers, biocontrol microbes, optimized microbiomes, soil amendments and matching microbe‐optimized crops for different soil types would be the ultimate goal for enhancing plant–microbe interactions. Clearly, this is a largely untapped area that deserves major research efforts, as it holds the promise to improve crop yields and address food security in an environmentally friendly and sustainable manner. Overall, existing microbial technologies along with emerging microbiome and associated approaches offer new and more sustainable practices to increase agriculture productivity. Initial assessments highlight growing demand for microbial‐based solutions for food security both from growers and consumers of the produce. However, significant scientific and technological challenges exist. If these challenges can be prioritized along with the improvement of regulatory framework (e.g. registration of products, safety requirements), emerging microbial‐based solutions can potentially transform sustainable agriculture. Given that agriculture has been central to the success of *Homo sapiens*, it is not surprising that such an approach can address multiple SDGs if implemented systematically.

## Conflict of interest

Matthew Wallenstein is a cofounder of Growcentia, Inc.
